# Transcoding number words to typed multi-digit numerals: revisiting the strange case in Arabic bilinguals

**DOI:** 10.1007/s00426-025-02097-1

**Published:** 2025-03-13

**Authors:** Maisam Hayek, Shoshi Dorfberger, Zohar Eviatar, Avi Karni

**Affiliations:** 1 The Edmond J Safra Brain Research Centre for the Study of Learning Disabilities, University of Haifa, Haifa, Israel; 2 Department of Learning Disabilities, University of Haifa, Haifa, Israel; 3 Special Education Department, Sakhnin Academic College, Sakhnin, Israel; 4 Gordon College of Education, Haifa, Israel; 5 Department of Psychology, University of Haifa, Haifa, Israel; 6 Institute for Information Processing and Decision Making, University of Haifa, Haifa, Israel; 7 The Sagol Department of Neurobiology, University of Haifa, Haifa, Israel

## Abstract

Language-specific effects are observed in the performance of numerical tasks. We evaluated the effect of the number word system (non-inverted; decade-unit format HDU vs inverted; unit-decade format HUD) in multi-digit numbers on transcoding performance among Arabic-Hebrew bilingual university students. Both languages are written right-to-left, but only in Arabic the sequence of number words is inverted, with units stipulated before decades (e.g. ‘four-and-twenty’). Previously we (Hayek et al., Writing Systems Research 11:188–202, 2019) addressed the effect of the number word system on transcoding by handwriting while in the present study, participants were asked to type the numerals. We found that the transcoding ability of verbally presented numbers in the standard inverted format, of bilingual adult native speakers of Arabic was inferior to their ability to transcode multi-digit number words in Arabic in a nonstandard format (HDU). These results were replicated in the present study. In addition, we found that native speakers of Arabic were actually most fluent in transcoding when the number words were presented in Hebrew, a language in which they had high proficiency, and were equal in transcoding ability when number words were presented in English, a language in which they were less proficient. The results suggest that even in a highly proficient bilingual adult, transcoding routines may be affected by the number word system regardless of language dominance.

## Introduction

The number word system refers to how numbers are coded in language-specific ways, including the names and syntax used in spoken language and their representation in writing systems (Pixner et al., 2011). A notable feature in many languages, including Maltese, German, Dutch, Danish, Malagasy, and Arabic and partially in Czech and Norwegian, is the inversion of decade and unit order (Comrie, 2005, 2006). In these languages, when expressing numbers from 21 to 99 verbally, the unit precedes the decade, creating an inversion compared to the written Arabic numeral notation (e.g., “eight-and-forty” represents 48). Research shows that for native speakers of languages with this inverted structure, this unit-decade inversion can impact task performance, particularly in calculations, leading to reduced speed and accuracy compared to languages where verbal and written numerical notations align (Helmreich et al., 2011).

The effect of number word inversion is particularly interesting in the context of bilinguals, where individuals must navigate between languages with different number word systems. Most models of bilingual comprehension propose that bilinguals activate both languages when processing words or sentences in any one of their languages (e.g., Brysbaert and Duyck, 2010; Kroll and Stewart 1994; Kroll et al., 2010). These models suggest that bilinguals share representations across languages (phonology, orthography, syntax) and that these representations become activated non-selectively. However, this may not hold true for how specific number processing mechanisms operate across languages. There is no clear evidence for cross-language influence when bilinguals process two-digit number words (Macizo et al., 2011). Number processing may be dependent on the language-specific format in which the names of the numbers are presented and other factors such as proficiency (Brysbaert et al., 1998; Fias, 2001; Garcia et al., 2021).

To better understand the interaction between the number word system and linguistic processing, we focus on transcoding- the conversion between verbal number words and written numerals. The ability to transcode indexes the relationship between specific mechanisms for processing visual numerals and verbal number words. Studies conducted to evaluate children's transcoding abilities (Barrouillet et al., 2004; Camos, 2008; Pixner et al., 2011; Power and Dal Martel, 1997, Zuber et al., 2009) suggest that the ordering of number words, specifically an inverted unit-decade format, affects transcoding performance, where children commit more errors than in languages where no inversion occurs. About half of the errors directly reflect the inversion (e.g., writing 24 instead of 42). Such inversion errors occur very infrequently among children using languages with noninverted number word system.

One factor that affects transcoding performance is the level of accumulated experience. Research shows that transcoding accuracy improves significantly with age and experience (Ganayim and Dowker, 2021; Zuber et al., 2009). Recent evidence directly demonstrates that the effects of unit-decade (UD) inversion on accuracy decrease during development, with adults showing nearly faultless performance (Cohen Kadosh and Walsh, 2009; Delazer et al., 2003; Ganayim and Dowker, 2021; Hayek et al., 2019).

However, while accuracy reaches ceiling levels in adulthood, the effect of inversion on processing speed remains less clear. This is particularly true for languages where inversion is characteristic of the number word system, such as German (Proios et al., 2002). Specifically, it remains unclear whether adults achieve the same level of transcoding fluency (speed) in languages with inversion compared to languages without inversion. Moreover, given that phonological representations and symbolic visuospatial representations are engaged in transcoding spoken number words to numerals (Camos, 2008; Pixner et al., 2011), transcoding procedures tax working memory capabilities. Studies (e.g., Barrouillet and Lepine, 2005; Camos, 2008; Hayek et al., 2025; Zuber et al., 2009) suggest that working memory capacity affects transcoding ability not only through the ability to remember the lexical elements and their sequence, but also through the ability to manipulate the sequence of digits in multiple formats and verbal notations.

The case of Arabic presents a unique opportunity to study these effects due to its linguistic structure and writing system. A number of studies suggest that the number word system in the Arabic language may pose a challenge to number processing and ascribe these difficulties at least in part to the inversion of the order of decade and units (Ganayim et al., 2020; Ganayim and Ibrahim, 2014, 2015; Hayek et al., 2019, 2025; Prior et al., 2015; Shaki et al., 2009). Transcoding from Arabic raises the same problem as transcoding from other languages where unit-decade inversion format exists. Additionally in Arabic, there is opposing writing direction for words and numerals. Thus, unlike, for example, in German (wherein 23 is “three and twenty” but text and numerals are written in the same direction, left to right), in Arabic, when numbers are written as words, the units come *before* (to the right of) the decade, but when numbers are written as numerals, schoolchildren are taught to write the digit designating the decade first. Thus, the units are again written to the right of the decade but *after* the decade because the numbers are written from left to right. Therefore, a double inversion is needed in transcribing multi-digit words in Arabic into numerals. First there is a mechanical shift from right to left (in producing the verbal written form) but switching to left to right for writing numerals. Second there is a cognitive switch (as in German) from units-decade (verbal) to decade-units (numerals). This can lead to uncertainty and confusion in ascribing the units and decades. Note that no such grounds for uncertainty are present in, for example, Italian or English.

Prior et al., (2015) investigated the effects of the number word system in Arabic on calculation and reported that adult Arabic-Hebrew bilinguals performed best, in simple addition, when numbers were presented auditorily in the standard format of the respective language (inverted UD in Arabic and not inverted DU in Hebrew). In the visual symbolic notation, there was no preference for different orders of numerals (5 + 20 = 25 vs. 20 + 5 = 25) in problem presentations in terms of either accuracy or speed. These findings suggest that the advantage of the standard format in each language (inverted UD in Arabic L1, noninverted DU in Hebrew L2) was observed only when numbers were presented as spoken words, and not when presented as digits. This suggests that the language-specific number word system affects processing only with verbal presentation.

A more recent study (Ganayim et al., 2020) investigated the effects of language-specific number word systems on transcoding performance in Arabic-Hebrew bilingual university students, who were native Arabic speakers proficient in Hebrew as a second language. Participants completed two tasks in both Arabic (L1) and Hebrew (L2): writing two-digit numbers to dictation (accuracy) and reading two-digit numbers aloud (Speed and accuracy). In this study, the stimuli in each language were presented in the characteristic format for that language. Performance speed and accuracy were better when processing numbers in Arabic (L1) compared to Hebrew (L2).

## The current study

This study examines transcoding proficiency in Arabic-Hebrew bilingual adults, focusing on the effect of unit-decade inversion present in Arabic but not in Hebrew, languages that differ in their number word system. The number words used were presented in two formats: inverted (Hundred-Units-Decade HUD) and “noninverted” (Hundred-Decade-units HDU). The current study followed a procedure and protocol identical to that that was used in our previous study (Hayek et al., 2019) exploring the effect of the number word system on transcoding in handwriting. The important difference between the two studies is that in the present study participants responded by typing. In the typing responses, decades must be typed before units. However, in our previous study, where participants wrote their response, they were free to write the decades and the units in any order they chose: hundreds, and then units with a gap in between to be filled by the tens or in the stipulated order that is taught at school—from left to right (hundreds, decades, units). In that study the majority of Arabic speakers left a gap between the hundreds and units in order to fill in the decades afterwards. This is not possible in the typing response. Hayek et al., (2019) reported slower performance in the inverted format. The goal of the present study is to examine accuracy and fluency when order cannot be flexible.

Only Arabic and Hebrew speakers were compared**.** This is a theoretically interesting comparison when we take writing direction seriously. Both languages are written from right to left and both require a change in direction when writing numbers. However, number inversion appears only in Arabic and not in Hebrew. Thus, experience with number inversion was the crucial difference between the language groups.

The design of our research allowed us to test several different issues. One was to compare transcoding abilities in two right-to-left languages (Arabic and Hebrew), where only Arabic features unit-decade inversion. In addition, because there have been contradictory findings (Hayek et al., 2019; Prior et al., 2015), the current study examines whether the linguistic structure affects processing efficiency more than language dominance. We also investigate how language proficiency in a non-native language (high in Hebrew, lower in English) affects number processing. We also varied input modality (auditory versus visual presentation) across languages. In our previous study (Hayek et al., 2019), there was no difference between Hebrew and Arabic speakers when numbers were presented as spoken words. However, Arabic speakers responded more slowly than Hebrew speakers to written words. We interpreted this as reflecting slower reading in Arabic than Hebrew that has previously been reported (Eviatar et al., 2019) due to the higher visual complexity of Arabic orthography.

Given the inversion of decade and units in Arabic and based on previous research (Hayek et al., 2019), we investigated whether the performance of native speakers of Arabic would be affected by the input format (UD inversion, DU no inversion), by language status (native vs. non-native) and modality (visual vs. auditory) when asked to transcode multi-digit number words to Arabic numerals by typing. The research questions and hypotheses are the following:

1. Since developmental studies show changes in the effects of number format with age on accuracy (Ganayim and Dawker, 2021), we test whether the effects of inversion will still be evident in processing speed.
2. The effect of input format: Since our previous study with handwritten responses showed advantages for noninverted formats (Hayek et al., 2019), we expect that Arabic speakers will respond faster to DU noninverted format and faster in Hebrew than Arabic, regardless of language status.
3. Since language proficiency affects numerical processing, we expect better performance in Hebrew (high L2 proficiency) compared to English (lower L2 proficiency), despite both being noninverting languages.
4. We expect an advantage for auditory over visual presentation, particularly in Arabic, due to the complexity of Arabic orthography. If we find this only in L1 (in Arabic for Arabic speakers but not in Hebrew for Hebrew speakers) this supports the hypotheses that the slowness of Arabic speakers is due the complex orthography of the writing system.

## Materials and methods

### Participants

The participants were native speakers of Arabic, and the control group comprised native speakers of Hebrew. All participants were students at the same university. Since all participants were adult university students who had passed the same entrance exams, we are quite confident that the differences we found are due to the differences in languages background and not of the education. The native speakers of Arabic were divided into two groups according to the languages tested: (1) Arabic and Hebrew conditions (N = 17; mean age = 25 years). (2) Arabic and English conditions (N = 16: mean age = 24 years). The participants in the control group, native speakers of Hebrew (N = 14; mean age = 24 years), who were not familiar with an L2 language that use UD inverted format in their number system, were tested only in the Hebrew number typing conditions.

All participants were recruited according to the following criteria:

1. Healthy, right-handed, above 18 years old, normal or corrected vision and hearing, with no chronic use of medications.
2. There was no history of learning disabilities or ADHD.
3. All had studied mathematics at school in their native language using Arabic numerals.
4. All of the participants passed the matriculation in Hebrew as well as a Hebrew and English language test as part of entrance test for the university.
5. All of the native speakers of Arabic were highly proficient in Hebrew as a second language, as it is the majority language in their everyday life and the main (in many cases the only) language of teaching in the university.

The study was approved by the Faculty of Education, University of Haifa Human Experimentation Ethics Committee (# 298/15). Participants were asked to confirm their consent to participate in the study after receiving an explanation of the experiment by signing the informed consent form. All participants received monetary compensation (40 NIS) for their participation.

### Stimuli and block design

The target stimuli consisted of two- and three-digit verbal numbers selected randomly from between 21 and 987. Excluded from the stimulus set were multiples of 10 (e.g., 20, 30, 40, 200, 300, etc.) and tie numbers (i.e., numbers for which there was an identity in the digits denoting hundreds, decades and units (e.g., **666**; **222**); hundreds and decades (e.g., **66**2; **55**7); decades and units (e.g., 3**66**; **55**); or hundreds and units (e.g., **3**6**3**).

### Procedure

All testing was conducted individually in a quiet room via a computer using an E-prime script (2.0; Psychology Software Tools, Inc. Pittsburgh, PA) and a standard keyboard. The experiment consisted of several conditions, with each condition presented as a separate block of trials. The conditions varied by:

1. Input modality:
2. Visual presentation (numerals or number words)
3. Auditory presentation (recorded by a male trilingual speaker with no accent in Arabic, Hebrew, or English)
4. Number format:
5. HDU (Hundred-Decade-Unit)
6. HUD (Hundred-Unit-Decade)

Each condition contained 30 trials with 7 two-digit numbers and 23 three-digit numbers. The numbers were randomized and never repeated within or across conditions. The two-digit numbers included in a given condition were not used to construct the corresponding three-digit number (e.g., if **53** was included, 6**53** was not presented). Preliminary analyses showed no relevant interactions between effects for the two- or three-digit numbers, so the data were pooled**.**

The native speakers of Arabic performed the task in Arabic and in the other language, Hebrew for Group 1 and English for Group 2. Order of language was counterbalanced across participants within the groups. Overall, each Arabic speaking participant faced 9 conditions. The native speakers of Hebrew were tested only in Hebrew for both visual and auditory condition. (i.e., 5 conditions). Examples of the 9 conditions presented in Table 1.

**Table 1:** Experimental Design: The 9 conditions presented for each participant in Group 1 and Group 2

Condition			
Input modality		Format	Example
1.	Visual digit		453
2.	Visual number words format (L1)	HUD	أربعمائة ثلاثة وخمسون
3.	Visual number words format (L2)	HUD	Group 1: ארבע מאות שלוש וחמישים Group 2: four hundred three and fifty
4.	Visual number words format (L1)	HDU	أربعمائة خمسون وثلاثة
5.	Visual number words format (L2)	HDU	Group 1: ארבע מאות חמישים ושלוש Group 2: four hundred fifty and three
6.	Auditory number words format (L1)	HUD	Spoken number words in Arabic
7.	Auditory number words format (L2)	HUD	Spoken number words in Hebrew for Group 1 and English for Group 2
8.	Auditory number words format (L1)	HDU	Spoken number words in Arabic
9.	Auditory number words format (L2)	HDU	Spoken number words in Hebrew for Group 1 and English for Group 2

Participants were asked to respond by typing Arabic numerals using the top row of a standard computer keyboard. Task instructions for each condition were followed by 3 practice trials to provide the participants with a clear understanding of what they were required to do during the ensuing condition.

In each condition, participants were instructed to ‘type each target number as digits as quickly and as accurately as possible’. At the beginning of each trial, a fixation cross appeared at the center of the screen, and participants were instructed to press the space bar of the keyboard to indicate their readiness to begin the trial. Seventeen msec after the space bar press, the fixation cross disappeared, and the target number was presented. All the responses were performed with the right hand. In the visual conditions, the stimuli were presented in the center of the screen, and disappeared (offset) at the beginning of the response. In the auditory conditions, participants were instructed to start responding only after hearing the complete (2-digit or 3-digit) stimulus because stimulus presentation was stopped whenever the writing response was initiated. No feedback on any of the performance measures was provided.

### Performance assessment and data analysis

Performance was assessed by using two measures. *The performance time* of correct responses constituted the total typing time, averaged across the blocks. Note that in the visual input condition, time was measured from the onset of the stimulus display, while in the auditory input condition, time was measured from the time of last digit onset. We could not analyze the responses from the two modalities in the same analysis because of differences in the timing of the stimulus presentations. We therefore present the analyses of responses per input modality. *The accuracy* was scored based on the number of errors in the output per block. In this analysis, modality was included as an independent variable.

## Results

### Background variables

Background variables are presented in Table 2. The participants were asked to rate their English language proficiency (reading, writing, listening comprehension, and speaking) using a self-report questionnaire (Macizo et al., 2011). In addition, the participants underwent two dictation tests to directly assess their proficiency in English number words: *i*. an auditory number dictation task (numbers 1–20 presented in a random order); and *ii*. a test of the accuracy and speed of voiced reading of visually presented number words (reading numbers 1–20 in a random order). Short term memory (STM) for digits was assessed using the WISC digit-span memory test (WISC-IV). There were no significant differences between the groups on any of these measures (Table 2).

**Table 2:** Characteristics of participants in the study: mean scores and standard deviation for self-ratings of English proficiency and tests, STM scores and years of education

	Native speakers of Arabic	Control group: Hebrew typing condition	t-test
English language proficiency questionnaire (self-rating 0–10)	5.61(1.72)	5.2 (1.6)	t(45) = − 0.7, p = 0.48, BF = 0.11
Dictation task in English (number of correct items)	20(0)	19.69(1.10)	t(45) = − 1.5, p = 0.15, BF = 0.001
Reading numbers in English (1–20) (time to completion, seconds)	11.02(2.46)	11.96(2.68)	t(45) = 1.09 p = 0.28, BF = 0.02
Reading numbers in English (1–20) (number of correct items, 20 items)	20(0)	20(0)	
STM (native language)	18.8(3.2)	18.85(3.26)	t(45) = 0.87 p = 0.39, BF = 0.095
Years of education	15.03(3.08)	13.71(1)	t(45) = − 1.56 p = 0.13, BF = 0.000

The test block, that assessed the basic digit typing speed with the stimuli to ensure that groups were comparable in terms of digit typing abilities showed no significant differences in basic digit typing speed among the 3 groups (*F*(2,45) = 1.90, *p* = 0.15).

### Transcoding performance in arabic

A within subjects t test was also conducted for the Arabic speaking participants, to test for differences in the performance of nonstandard HDU Arabic compared to that of the standard HUD format of Arabic. This comparison was run on all participants (N = 33) because both groups were tested in Arabic (their native language).

**Fig. 1:**
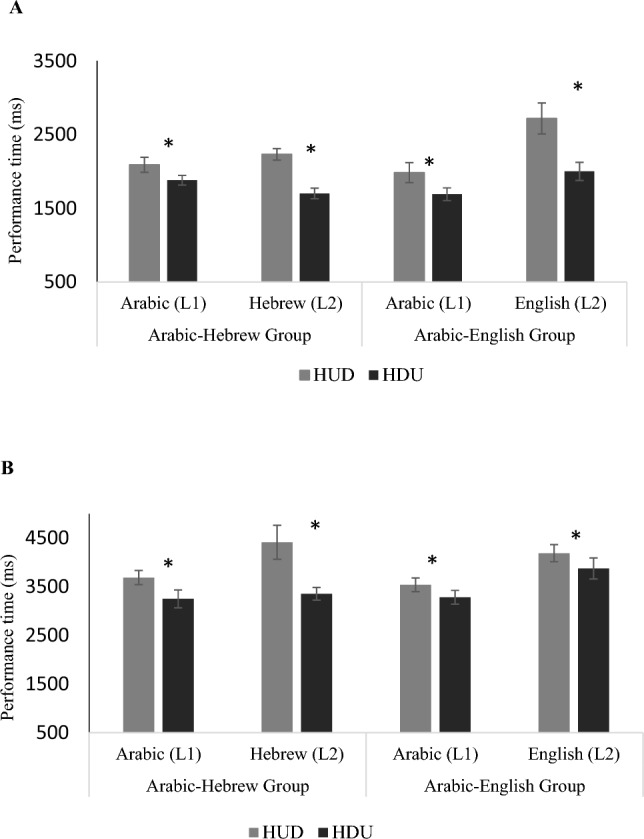
Means and SDs of performance time among native speakers of Arabic in the auditory (panel **A**) and visual (panel **B**) conditions. *HUD* hundreds, units followed by decade (the standard in Arabic); *HDU* hundreds, decades, units (the standard in Hebrew and English)

There was a clear advantage in terms of performance times for typing multi-digit numbers presented in the HDU format compared to the HUD format, both in the auditory (paired *t*(32) = 4.45,* p* < 0.001, *d* = 1.57) and visual (paired *t*(32) = 3.62,* p* < 0.001, *d* = 1.24) modalities (Fig. 1A, B). Thus, native speakers of Arabic performed better when presented with numbers in the HDU, nonstandard Arabic format, than when presented with numbers in the HUD standard Arabic format.

The accuracy of performance was not affected by the format (HDU or HUD) in which the numbers were presented. On average, participants tended to commit few errors, and there were no significant differences in the number of errors committed when the target numbers were presented in the noninverted (HDU: auditory: M = 15%, SD = 3.8; visual: M = 27.06%, SD = 5.2) vs. inverted (HUD: Auditory: M = 18.3%, SD = 5.8; Visual: M = 29.16%, SD = 6.7) format (*t*(32) = 0.81,* p* = 0.43, *d* = 0.28; *t*(32) = 0.38,* p* = 0.71, *d* = 0.13 auditory and visual modality, respectively). For the auditory modality Bayes factor (0.21) indicated a higher probability that there really was no difference between the inversion conditions. For the visual modality, Bayes factor approached this cutoff (0.35). Thus, our participants did not show an inversion effect in the very few errors they made.

### Comparing performance between inverting (Arabic) and noninverting (hebrew/english) languages in Arabic speakers

#### Performance times

The data from the participants who were tested in Arabic and Hebrew (Arabic-Hebrew group) and those tested in Arabic and English (Arabic-English group) were analyzed to determine the differences in performance times when the inverted or noninverted target numbers were presented in either Arabic or in a noninverting second language. Given that our participants were highly fluent in Hebrew but somewhat less fluent in English, this analysis allowed us to test the effects of language proficiency on number processing. To this end, a repeated-measures ANOVA was run with Language (native, second-L2) and Format (HDU, HUD) as within-subject factors and Group (Arabic-Hebrew, Arabic-English) as a between-subjects factor. The analysis was run separately for the auditory and the visually presented number words.

There was a significant main effect of Language, with an advantage for typing numbers presented in Arabic (L1) compared to L2 (Hebrew/English) in both the auditory modality (*F*(1,31) = 20.13,* p* < 0.001, *partial η*^*2*^ = 0.05) and in the visual input modality (*F*(1,31) = 41.64,* p* < 0.001, *partial η*^*2*^ = 0.09). There was also a significant main effect of Format in both input modalities (auditory: *F*(1,31) = 72.23,* p* < 0.001; *partial η*^*2*^ = 0.15; visual: *F*(1,32) = 43.7,* p* < 0.001; *partial η*^*2*^ = 0.09), with an overall advantage in typing number words in the HDU format (across both the native and second language) compared to HUD format across both the native language (Arabic) and second language (Hebrew/English). Importantly, in the auditory input modality, there was a significant interaction of Language × Group (*F*(1,31) = 24.9, *p* < *0.0*001; *partial η*^*2*^ = 0.06). As shown in panel A of Fig. 1, planned comparisons showed that this interaction is due to the fact that in Group 1 the responses in Hebrew (L2) were as fast as the responses in Arabic(L1) *F*(1,31) = 5.26, *p* = 0*.*03). While In group 2, the responses in English (L2) were significantly slower than in Arabic (*F*(1,31) = 15.11, *p* = 0*.*0015; *partial η*^*2*^ = 0.02).

Moreover, there was a significant interaction for Language × Format (*F*(1,31) = 21.44,* p* < 0.001; *partial η*^*2*^ = 0.03). Panel A of Fig. 1 also shows that the effect of Format in the auditory condition was larger in L2 (Hebrew/English) than in Arabic in both groups; i.e., the costs of inversion were larger in Hebrew and English, both noninverting languages, than in Arabic.

In the visual input modality, there was also a significant interaction of Format × Group (*F*(1,31) = 8.6, *p* < 0.006; *partial η*^*2*^ = 0.02). As shown in panel B of Fig. 1, although the effects (costs) of the unit-decade inversions were significant under all of the conditions, they were largest in Hebrew. No other significant interactions were found in the visual modality.

To more closely examine the effects of language proficiency on inversion, we compared performance in the standard format of Arabic (HUD) and either the standard (HDU) format in Hebrew or, separately, (HDU) in English. A comparison with Hebrew revealed an advantage for transcoding stimuli in Hebrew in both the auditory (paired *t*(16) = 5.4,* p* < 0.001, *d* = 2.61) and visual (paired *t*(16) = 3.36,* p* < 0.01, *d* = 1.62) modalities. Thus, native Arabic speakers typed number words more efficiently when they were presented in the standard format in Hebrew, an L2, than in their native Arabic standard format. For the group that responded to English as L2: In the visual modality, performance was slightly faster in Arabic than in English (paired *t*(15) = 2.23, *p* = 0.04, *d* = *1.15*). However, in the auditory modality, the comparison with performance in English revealed no significant difference (*p* > 0.8). Thus, even in a language in which they were less proficient, but is noninverting, the Arabic speakers in our sample typed as fast as they did when transcoding in their native language.

Moreover for the group that responded for Hebrew as L2: when we compared performance between noninverted Arabic (nonstandard, HDU) and standard Hebrew (HDU) languages, there was an advantage for standard Hebrew in the auditory input modality (paired *t*(16) = 3.31, *p* < 0.01, *d* = *0.64*), and there was no significant difference in performance time in the visual input modality (paired *t*(16) = 0.77, *p* = 0.45). Thus, native speakers of Arabic performed better in standard Hebrew even than in nonstandard Arabic (in 2 conditions having the same order of digits).

To further examine format effects across languages, we compared performance in the HUD format between Arabic and Hebrew, no significant differences were found in either auditory (paired *t*(16) = − 1.104, *p* = 0.28), or visual (paired *t*(16) = − 1.92, *p* = 0.064) modalities. These findings suggest that when the format structure (HUD) is held constant across languages, performance patterns are comparable regardless of language status (L1/L2).

#### Error scores

**Fig. 2:**
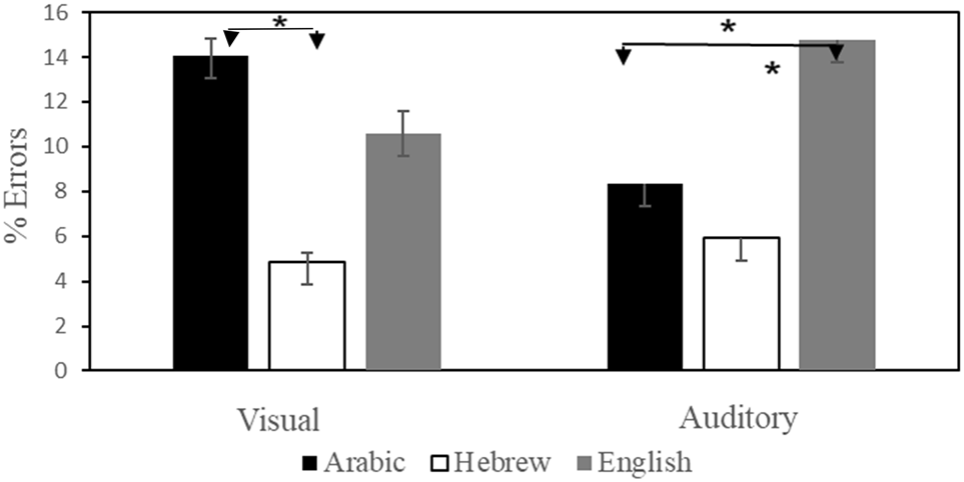
Percentage of errors (standard errors) for native speakers of Arabic in Arabic, Hebrew and English for the auditory and visual modality

A repeated-measures ANOVA with Language (native, Hebrew, English), Format (HDU, HUD) and Modality (Visual, Auditory) as within-subject factors, and Group (Arabic-Hebrew, Arabic-English) as a between-subjects factor showed a significant Language effect, with an advantage for typing numbers presented in Hebrew (L2) compared to Arabic (*F*(1,31) = 12.15,* p* < 0.01, *partial η*^*2*^ = 0.06) and compared to English* F*(1,31) = 14.32, *p* < 0.01, *partial η*^*2*^ = 0.08). There was also a significant interaction of Modality × Language (*F*(1,31) = 9.3,* p* < 0.001; *partial η*^*2*^ = 0.05). As shown in Fig. 2, this interaction occurred because participants tended to make significantly more errors in Arabic than in L2 Hebrew in the visual input modality (*F*(1,31) = 17.61,* p* < 0.01, *partial η*^*2*^ = 0.16) while in the auditory input modality, participants made significantly more errors in English than in Arabic (*F*(1,31) = 10.97,* p* < 0.01, *partial η*^*2*^ = 0.08) with no differences between Arabic and Hebrew (*F*(1,31) = 1.57,* p* = 0.214). In Arabic, participants made significantly more errors in the visual input modality than in the auditory input modality (*F*(1,31) = 14.82,* p* < 0.01, *partial η*^*2*^ = 0.81). No such significant differences were found between the two input modalities in English (visual:* F*(1,15) = 3.06,* p* = 0.08) or Hebrew (visual: *F*(1,16) = 0.86, *p* = 0.36).

Correlations between number of errors and performance speed were computed and none indicated speed accuracy tradeoffs.

### Comparison of native speakers of Arabic to native speakers of Hebrew: the transcoding of number words and the typing of digits

**Fig. 3:**
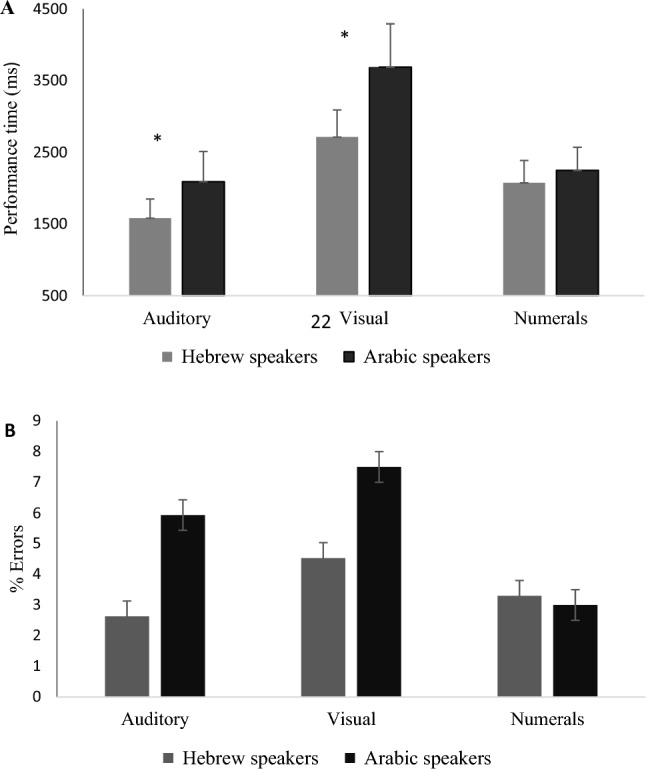
Performance time (Panel **A**) and percentage of errors (Panel **B**) in the participant’s native language for Arabic and Hebrew speakers in the two visual (numerals, number words) and the auditory condition

Figure 3 presents the comparative data on the performance of native speakers of Arabic and native speakers of Hebrew when stimuli were presented either as digits (Arabic numerals, presented visually) or as number words in the standard format of each language. The participants in the two groups were matched for age, education, verbal working memory capacity and English proficiency, as shown in Table 2. A repeated-measures ANOVA was run on performance time for the visual conditions, with Task condition (numerals, number words) as a within-subjects factor and native Language (Hebrew, Arabic) as a between-subject factor. There was a significant difference in performance times between task conditions (*F*(1,29) = 206.6,* p* < 0.001; *partial η*^*2*^ = 0.88) with slower typing in the number words condition and a significant effect of native language with an overall advantage for the native speakers of Hebrew (*F*(1,29) = 17.81,* p* < 0.001; *partial η*^*2*^ = 0.38). However, there was also a significant interaction of Task condition × native Language (*F*(1,29) = 30.72,* p* < 0.001; *partial η*^*2*^ = 0.51). This interaction occurred because there were no significant differences in language performance time when multiple digits were presented onscreen as printed Arabic numerals (*t*(29) = − 1.52,* p* = 1.4). Bayes factor (0.008) indicated a higher probability that there really was no difference between the groups. Thus, the 2 groups were equal in terms of the typing of the numbers presented (visually) as digits. However, a significant disadvantage was found for native speakers of Arabic compared to their Hebrew speaking peers when the items were presented as number words (t(29) = 5.25,* p* < 0.001, *d* = 1.89).

In terms of accuracy, a parallel ANOVA showed a significant effect of Task (*F*(1,29) = 5.66,* p* < 0.05; *partial η*^*2*^ = 0.16), with lower accuracy when number words were visually presented compared to when digits were presented. There was no significant effect of native Language (*F*(1,29) = 1.03, p = 0.32). There is strong evidence in favor of an absence of effect of native Language, with Bayes factor for words = 0.008; and marginal evidence for digits, Bayes factor = 0.33) and no significant interaction of Task condition x native Language (*F*(1,29) = 1.92,* p* = 0.18).

In the auditory modality, a comparison of the performance speed of the two groups when number words were presented in their respective native languages showed a significant advantage for native speakers of Hebrew (unpaired *t*(29) = − 3.91,* p* < 0.001, *d* = 1.4). The number of errors committed was not significantly different between the Language groups (*t*(29) = 1.02, p = 0.32, d = 0.37). Bayes factor (0.00) indicated a higher probability that there really was no difference.

## Discussion

The present study investigated the effects of language-specific number word systems on transcoding performance in Arabic-Hebrew bilingual adults. Our findings reveal complex interactions between linguistic structure, language proficiency, and cognitive processing in numerical tasks. We will discuss our results, focusing on three key areas: (1) persistence of inversion effects in adulthood, (2) bilingualism and language-specific effects on number processing (3) modality differences in transcoding.

### Persistence of inversion effects in adulthood

Our results challenge the assumption that the effects of UD inversion disappear in adulthood due to increased experience and skill. Previous studies, especially those focusing on children, reported on accuracy of responses and indeed our accuracy results do not show an effect of inversion (e.g., Bahnmueller et al., 2015, 2021; Zuber et al., 2009). However, in both this study and our previous study (Hayek et al., 2019), we found that young adult native speakers of Arabic, with life-long exposure to the UD inversion, consistently performed faster when transcoding number words presented in a HDU format compared to the HUD inverted standard Arabic. Importantly, while in our previous handwriting study (Hayek et al., 2019) participants could adapt to inversion by employing flexible strategies (such as leaving spaces between digits), the typing task in the current study required strict left-to-right sequential input. This methodological constraint revealed that when compensatory strategies are not available, the impact of inversion persists in processing speed. The persistence of inversion effects even with typing suggests these effects remain robust in skilled adults. This advantage for the HDU format was observed in both visual and auditory modalities. The benefit from noninversion of the number name was larger than the benefit of grammaticality (standard format) and larger than the benefit of using one’s native tongue (responses in Hebrew in L2 were faster than responses in Arabic). Our findings align with other studies in adults, showing that the UD format may still exert costs mainly in terms of speed (Brysbaert et al., 1998; Hayek et al., 2025; Lonnemann and Yan, 2015; Prior et al., 2015).

### Bilingualism and language-specific effects on number processing

The comparison of performance of our Arabic speakers in two second languages can inform us about the effect of second language proficiency and language-specific effects on transcoding. Both of the second languages in which we tested our participants do not have number inversion. However, the comparisons with Hebrew and English present different effects. We believe that this is a reflection of a combination between proficiency in L2 and language-specific effects. Our participants were highly proficient in Hebrew. As shown in Figs. 1 and 2, they responded more quickly and more accurately in Hebrew than in Arabic. In the auditory condition they responded faster in Hebrew than in Arabic in the HDU condition, where in the visual condition they responded more slowly in Hebrew in the HUD condition than in Arabic. Performance cost in speed of inversion overall, was higher in Hebrew (L2) than in Arabic (L1). Because of the high proficiency of our participants in L2 we believe that the results in both Arabic and Hebrew are due to the inversion effect. Our participants were less proficient in English. They performed more quickly in L1 than in L2. Overall, the performance was equivalent between the languages with an exception that HUD took longer in English in the auditory condition. We believe that the similar performance patterns in this case are due to different sources. In Arabic we are seeing the effect of inversion whereas in English we are seeing the effects of proficiency.

These results can be interpreted as an indication that native speakers of Arabic, bilinguals with high proficiency in L2 (Hebrew), may not develop a strong preference for their native language in the domain of number name representation. Maczio et al. (2011) suggest that acquisition of a second language may bias the processing of numerical cognition: when bilinguals are highly proficient in their L2, this proficiency can affect their L1 (in addition to the effects of L1 on L2) (Kroll et al., 2015).

It has been suggested that bilinguals process numbers differently from monolinguals and are less affected by the lexical properties of their languages (Macizo et al., 2011). From this perspective, the current results indicate that Arabic speakers do not transcode numbers presented in a noninverting L2 (DU) by translating numbers into the inverted Arabic format (UD) but process multi-digit number words in each language separately. Our findings offer new insights into how bilinguals manage potentially conflicting number representation systems. The fact that our Arabic-Hebrew bilingual participants performed better in Hebrew than in Arabic for comparable number formats, suggests that they have developed separate, efficient processing routes for each language, rather than relying on a single, language-independent system. Moreover, our findings indicate that extensive experience with a second language can significantly impact number processing in the first language. This bi-directional influence supports the idea that bilingual language processing is highly interactive, with proficiency in one language affecting performance in the other (Kroll et al., 2015). Our results provide strong evidence for language-specific effects in number transcoding processes, and may support the notion that numerical representations are not entirely abstract (Cohen Kadosh and Walsh, 2009).

Typing numbers that are presented in standard grammatical format in Arabic is harder that in Hebrew. We conjecture that this may be due to the inversion load that is unique to Arabic (compared to Hebrew and English). Typing HUD number words in Arabic requires inversion of the units and decades in multi-digit numbers. i.e., entering in a strict order the decade first and then the unit which incongruent with the grammatical linguistic presentation. Furthermore, native speakers of Arabic were as fast at typing multi-digit numbers presented in HUD nonstandard Hebrew compared to standard HUD Arabic. Numbers in HUD format, even though standard in Arabic and novel in Hebrew, were transcoded with similar speed and accuracy in the two languages. This pattern of results can be taken as an indication that *familiarity* with the inverted format may not be the main factor affecting the transcoding of number words, but rather the cognitive cost of inversion. Given our results, we speculate that high fluency in a second noninverting language may decrease the advantages of familiarity with the HUD inverted number format in the first language, especially in the auditory modality, in the context of number transcoding. It has been proposed that cognitive processing in bilinguals is determined by the attributes of both languages (Martín et al., 2010). The current results underscore the complex interaction and the combined importance of the linguistic format and the frequency of use (exposure) of the two languages in bilingual individuals.

In the study of Prior et al., (2015), bilingual participants were asked to indicate whether arithmetic statements (addition) were correct or not. In one condition they heard the statements in L1 and in L2, and in the other they saw digits. They did not find a significant effect of inversion, but overall, participants responded better in L1 than in L2. In our results we did not find an advantage of L1 on L2 and strong effect of inversion. We believe that this difference may be due to the different tasks demands.

### Modality differences in transcoding

In the control condition of typing multi-digits from a numerical representation (Arabic numerals), there was no difference between the native speakers of Arabic and native speakers of Hebrew. This pattern of results was also found in our previous study (Hayek et al., 2019). Differences between speakers in different languages occurred only when words representing number names were involved, placing this effect in the language system and not in number cognition. The two groups also were comparable in terms of education, proficiency in typing and verbal STM. But, in both the visual and auditory input modalities, native speakers of Hebrew performed better (in terms of speed) in their native language than native speakers of Arabic in their own native language. Diglossia (the gap between the spoken vernacular and the standard literary Arabic) probably does not play a role in our findings, as the words used to name numbers are nearly identical in the spoken and literary versions of Arabic (Saiegh-Haddad, 2003). The finding that the cost of inversion was stronger in the visual than the auditory modality may support the hypothesis that Arabic orthography is more complex and harder to decode than Hebrew orthography (Eviatar and Ibrahim, 2014; Eviatar et al., 2019).

### Limitations and future directions

While our study provides valuable insights into bilingual number processing, it has two main limitations that future research should address:

1. Sample Characteristics; Our study had a relatively small sample. In addition, the demographic measures we used were somewhat shallow and did not allow us to measure individual differences in an interesting way.
2. Task Specificity; we focused on one task. Future research should investigate whether similar language-specific effects are observed in other numerical tasks, such as arithmetic problem-solving or magnitude comparison.

## Conclusions

The transcoding ability of bilingual young adults, native speakers of Arabic (a language that stipulates units before decades, HUD), was inferior to their ability to transcode multi-digit number words in Arabic in a nonstandard format (HDU); bilingual native speakers of Arabic were actually most fluent in transcoding when the number words were presented in Hebrew (L2, HDU) compared to their performance in Arabic (L1, HUD). The results lend support to the notion that the processing of numbers and, specifically, transcoding routines, may be language-specific; transcoding performance is dependent on the language, task demands and the notation (words, numerals, input modality) used in the presentation of the target multi-digit numbers. The current results suggest, therefore, that typing numerals reflects a complex interplay between language-specific routines set to transcode verbal number words in a HUD inverted format, the specifics of the language and its orthography (including word writing and numeral writing direction) and the familiarity (level of experience) in noninverting languages (L2); all these factors may bring about significant effects on transcoding in Arabic that are experienced by Arabic-speaking multilinguals even as adults. This finding is particularly striking because it goes against the typical expectation of L1 advantage in cognitive tasks. It suggests that the linguistic structure of number words (HUD vs. HDU) can have a stronger influence on processing efficiency than language dominance or age of acquisition.

## Data Availability

The data that support the findings of this study are available from the corresponding author upon reasonable request.
